# Intra- and inter-tester reliability of post-occlusive reactive hyperaemia measurement at the hallux

**DOI:** 10.1186/1757-1146-8-S2-O1

**Published:** 2015-09-22

**Authors:** Alex Barwick, Sean Lanting, Vivienne Chuter

**Affiliations:** 1 Department of Podiatry, University of Newcastle, NSW, Australia

**Keywords:** Microvascular dysfunction, Peripheral arterial disease, Reliability, Post-occlusive reactive hyperemia

## Background

Post-occlusive reactive hyperaemia (PORH) is a measurement of the vasodilatory capacity of the microvasculature that is associated with cardiovascular disease, peripheral arterial disease and foot ulceration. Reliability of its measurement in the toe for clinical and research purposes has not been adequately assessed. This study assesses both the intra and inter-tester reliability of four methods of assessing PORH in the toe.

## Methods

A within-subject repeated measures design was used. Forty-two participants underwent PORH testing using four methods: pressure measurement with photoplethysmography; an automated laser Doppler technique with local heating; an automated laser Doppler technique without local heating; and a manual laser Doppler technique. Participants underwent testing on two occasions with a three to 14 day interval. Intra-class correlation coeficients and limits of agreement were used to assess reliability.

## Results

Laser Doppler measurement with a heating probe was found to be the most reliable method of PORH measurement. Index of the area under the curve of pre- and post-occlusion and peak perfusion as a percentage of baseline were the most reliable variables.

## Conclusion and clinical relevance

PORH can be reliably measured using laser Doppler when combined with a heating probe. Further research is required to determine clinical utility of photoplethysmography in the measurement of PORH as a measure of microvascular dysfunction in the periphery.

